# Causal inference of CLEC5A and ISG20 in atherosclerosis: integrating Mendelian randomization and eQTL evidence

**DOI:** 10.3389/fimmu.2025.1644135

**Published:** 2025-12-03

**Authors:** Ya Zhang, Qian Tian, Yuan Zhu, Tianhui Wang, Yongchao Yu, Lan Wang, Lijuan Shao, Xiangang Mo

**Affiliations:** 1 Comprehensive Ward, The Affiliated Hospital of Guizhou Medical University, Guiyang, China; 2 Department of Geriatrics, The Affiliated Jinyang Hospital of Guizhou Medical University, Guiyang, China

**Keywords:** atherosclerosis, Mendelian randomization, expression quantitative trait locus (eQTL) analysis, CLEC5A, ISG20, macrophages

## Abstract

**Introduction:**

Atherosclerosis (AS) is a vascular disorder characterized by lipid accumulation and chronic inflammation, with pathogenesis closely linked to genetic factors and immune regulatory mechanisms.

**Methods:**

This study comprehensively identified ASassociated genes by integrating data from the Gene Expression Omnibus (GEO) database and expression quantitative trait locus (eQTL) analyses, complemented by Mendelian randomization (MR) analysis, followed by experimental validation of their functional roles.

**Results:**

Results indicated significant upregulation of CLEC5A and ISG20 in patients with AS, with MR analysis revealing positive causal relationships between both genes and AS risk (CLEC5A: OR = 1.001, P = 0.047; ISG20: OR = 1.001, P = 0.030), while HOXA2 showed a negative causal association. Functional enrichment analysis highlighted CLEC5A and ISG20’s involvement in immune responses, inflammatory pathways, and lipid metabolism regulation. Experimental validation in oxidized low-density lipoprotein (ox-LDL)-stimulated macrophages and apolipoprotein E-deficient (ApoE^–/–^) mouse models consistently demonstrated significant upregulation of ISG20 expression (Western blot and RT-qPCR, P < 0.01). Immunofluorescence co-staining and immunohistochemistry confirmed its elevated expression in endothelial cell- and macrophage-rich regions of AS plaques.

**Discussion:**

This study represents the first to elucidate the molecular mechanism by which ISG20 promotes AS progression through macrophage lipid accumulation and inflammatory responses, positioning it as a potential novel therapeutic target for AS.

## 1 Introduction

Atherosclerosis (AS) is a chronic inflammatory vascular disease characterized by lipid accumulation in the arterial wall and infiltration of inflammatory cells, leading to vascular stenosis and atherosclerotic plaque formation (1). This pathological process is the primary cause of cardiovascular diseases (CVD), which remain the leading contributors to global mortality and disability (2). Despite advances in research, the pathogenesis of AS remains poorly understood, driven by complex interactions between genetic susceptibility and environmental factors. Therefore, the systematic identification of molecular regulators controlling atherosclerotic plaque dynamics is crucial for the development of targeted therapies.

Recent research has highlighted significant progress in understanding AS, particularly regarding immune mechanisms. Various immune cells, including macrophages, T cells, and dendritic cells, play pivotal roles in disease progression (3). Inflammatory biomarkers, such as C-reactive protein (CRP) and interleukins (ILs), have demonstrated clinical value in the diagnosis and prognostic assessment of AS (4). Pathophysiologically, considerable attention has been focused on the role of inflammatory responses in driving disease progression. Specifically, immune cell activation and the release of inflammatory cytokines have been shown to directly contribute to plaque destabilization (5). Additionally, advanced technologies like single-cell RNA sequencing (scRNA-seq) have revealed macrophage and monocyte heterogeneity within atherosclerotic lesions, offering transformative insights into disease mechanisms (6).

Genetic factors are critical in AS pathogenesis. Studies have identified specific genetic polymorphisms that contribute to disease development. Notably, variants in the proprotein convertase subtilisin/kexin type 9 (PCSK9) gene influence low-density lipoprotein cholesterol (LDL-C) metabolism, significantly increasing the risk of AS (7). Emerging epigenetic research has highlighted the central role of DNA methylation and histone modifications in AS progression. Dysregulated DNA methylation patterns, including promoter hypermethylation of anti-inflammatory genes and global hypomethylation in pro-atherogenic regions, are mechanistically linked to disease progression (8). These epigenetic changes disrupt transcriptional homeostasis, driving atherosclerotic plaque progression through the sustained activation of pro-inflammatory pathways.

Recent advancements in genetic analytical technologies have provided powerful tools for investigating the genetic basis of AS. Expression quantitative trait loci (eQTL) analysis, a cornerstone of functional genomics, systematically identifies cis-regulatory variants that modulate transcriptional activity (9). This method links non-coding SNPs to disease etiology by identifying causal genetic polymorphisms that affect mRNA abundance through transcriptome-wide association studies (TWAS). Mendelian randomization (MR) leverages genetically proxied instrumental variables (IVs) based on Mendelian inheritance principles to establish causal relationships between modifiable exposures and disease outcomes. By mitigating residual confounding and reverse causation biases prevalent in observational studies, MR analysis relies on three core assumptions: genetic variant-exposure association, exclusion restriction, and independence from pleiotropic pathways (10).

The Gene Expression Omnibus (GEO) database serves as a crucial resource for gene expression data, offering detailed sample metadata for researchers. By integrating eQTL analyses with MR, this platform enables the discovery of AS-associated genes within large-scale genomic datasets. These integrated strategies not only clarify the genetic architecture of disease pathogenesis but also provide novel mechanistic insights for translational research and therapeutic development. Through the combination of GEO-derived eQTL data with MR frameworks, CLEC5A and ISG20 were identified as susceptibility genes with genetically proxied causal associations to AS. These candidate biomarkers show potential as clinically actionable diagnostic tools, with their encoded proteins emerging as possible therapeutic targets. This dual-functional approach presents innovative opportunities for precision risk stratification and targeted interventions in atherosclerotic CVD.

## 2 Materials and methods

### 2.1 GEO data collection

Gene expression datasets related to AS were obtained from the GEO database (https://www.ncbi.nlm.nih.gov/geo/). The search strategy employed the keywords “atherosclerosis” and “plaque,” with species restricted to Homo sapiens. Datasets were selected based on the following inclusion criteria: a minimum sample size of 20 per dataset, availability of both raw and normalized high-quality data, a comparative experimental design with case groups (patients with AS) and control groups (healthy donors or non-atherosclerotic subjects), and microarray-based expression profiles with platform-matched probe annotation files.

### 2.2 Data preprocessing and DEG identification

Raw data from the datasets GSE9820, GSE34822, GSE40231, and GSE100927 were processed using R software (version 4.3.2). Initial preprocessing included batch effect correction for each dataset. Afterward, the four datasets were merged, and batch effects were further minimized using the ComBat function from the sva package. High-dimensional data were reduced to a three-dimensional space through principal component analysis (PCA) for visualization. Differentially expressed genes (DEGs) were identified using the limma package, with significance thresholds set at a false discovery rate (FDR)-adjusted P-value < 0.05 and absolute log_2_fold change (log_2_FC) > 0.585 (equivalent to ~1.5-fold change). Differential expression patterns were visualized using volcano plots and heatmaps.

### 2.3 Preparation of exposure data (gene eQTL data)

To identify genetic variants associated with gene expression, summary-level gene eQTL data from the GWAS Catalog (https://gwas.mrcieu.ac.uk/) were used as exposure data, derived from European ancestry populations. The R package “TwoSampleMR” was utilized to select strongly associated single-nucleotide polymorphisms (SNPs) meeting the genome-wide significance threshold (p < 5e-08) as IVs. Linkage disequilibrium (LD) clumping was performed with parameters of r^2^ < 0.001 and a window size of 10,000 kb, excluding SNPs within 10,000 kb that exhibited LD (r^2^ < 0.001) with the index SNP. SNPs with weak instrument bias (F-statistic < 10) were excluded to ensure robust causal inference.

### 2.4 Outcome data preparation

Outcome data were obtained from the IEU OpenGWAS database (https://gwas.mrcieu.ac.uk/), which provides genome-wide association study (GWAS) summary statistics. The specific GWAS dataset (ID: ukb-d-I9_CORATHER) included 361,194 samples, with 346,860 controls of European ancestry, and comprised 13,586,589 SNPs. All GWAS summary statistics used in this study are publicly available and freely downloadable. Ethical approval for this study was waived as it relied solely on de-identified, aggregate-level genetic data from pre-existing resources.

### 2.5 Mendelian randomization analysis

MR analyses were conducted using the “TwoSampleMR” R package. Five MR methods were employed: MR-Egger, Weighted median, Inverse-variance weighted (IVW), Simple mode, and Weighted mode, to infer causal relationships between genetic instruments and atherosclerotic outcomes. Genes associated with AS were identified through a three-step filtering strategy: IVW-derived P < 0.05; concordant odds ratio (OR) directions across all five methods; and removal of genes exhibiting horizontal pleiotropy (P < 0.05 in the MR-Egger intercept test or MR-PRESSO global test). Genes that passed these filters were considered robustly associated with AS pathogenesis.

### 2.6 Identification of overlapping genes

Key genes associated with AS were identified by intersecting the results from differential expression analysis and MR analysis. Specifically, upregulated genes were cross-referenced with high-risk genes (OR > 1), while downregulated genes were intersected with low-risk genes (OR < 1), resulting in overlapping gene sets for both upregulated and downregulated genes. These intersection results were visualized using Venn diagrams to highlight shared genetic signatures between the two analytical approaches.

### 2.7 Mendelian randomization analysis of overlapping genes

The overlapping genes identified through this cross-analysis—comprising both upregulated and downregulated genes—were further analyzed using MR to determine their causal relationships with atherosclerotic outcomes. In these MR analyses, the overlapping genes were treated as exposures, and disease status (AS) was modeled as the outcome. The analytical framework included: heterogeneity assessment via Cochran’s Q test; evaluation of horizontal pleiotropy using MR-Egger intercept and MR-PRESSO global tests; and sensitivity validation through leave-one-out analysis to identify influential SNPs. The robustness of causal estimates was confirmed through consistency across MR methods (IVW, weighted median, MR-Egger). Results were visualized through scatter plots (showing exposure-outcome associations), forest plots (illustrating effect size consistency), and funnel plots (depicting symmetry of genetic instrument effects).

### 2.8 GO and GSEA functional annotation

Gene Ontology (GO) functional annotation was performed to elucidate the functions and biological pathways of key genes. The identified genes were subjected to GO enrichment analysis using Bioconductor packages, including clusterProfiler, to pinpoint potential functional pathways and pathogenic mechanisms. Enrichment significance was set at an adjusted P-value < 0.05.

Gene Set Enrichment Analysis (GSEA) was used to assess whether predefined biological functions or pathways associated with key genes were enriched at the top or bottom of the ranked gene list, reflecting upregulated or downregulated trends, respectively. GSEA further explored the activity levels of related functions or pathways within transcriptomic profiles. A nominal P-value < 0.05 and an FDR < 0.25 were applied to identify statistically enriched gene sets.

Given the relatively limited size of the gene set investigated in this study, the findings should be regarded as hypothesis-generating and lack formal statistical validation for pathway enrichment. Consequently, the biological plausibility and potential mechanisms underlying these gene associations were examined.

### 2.9 Immune cell infiltration analysis

The relative abundance of immune cell subtypes within each sample was quantified using computational deconvolution algorithms. Specifically, the CIBERSORT algorithm in R was applied to transcriptomic profiles to estimate immune cell proportions. CIBERSORT utilizes a predefined reference gene expression signature matrix representing 22 immune cell types, including seven T-cell subsets (e.g., naive, memory, regulatory), B-cell lineages (naive B cells, memory B cells, plasma cells), natural killer (NK) cells, and myeloid subpopulations. Permutations (n = 1000) were performed to validate the robustness of the deconvolution results. The output provided the relative proportions of each immune cell type across samples, which were visualized using stacked bar plots to depict inter-sample heterogeneity in immune infiltration. Additionally, Pearson correlation analysis was conducted to explore associations between immune cell abundances and key gene expression levels, with the results visualized through heatmaps and correlation matrices.

### 2.10 Validation cohort differential analysis

Raw data from dataset GSE28829 were processed and quality-controlled using R software (version 4.3.2). Differential expression analysis was performed to compare key gene expression between the control and experimental groups, validating their association with disease. Validation results were systematically cross-checked with previous MR findings to assess consistency. Statistical significance was determined using Student’s t-test (adjusted P < 0.05) and effect size concordance (logFC).

### 2.11 Experimental validation

#### 2.11.1 Experimental animals

Six mice, aged 5–8 weeks, including three ApoE^–/–^ mice (provided by Dr. Hailong Ou and commercially sourced from Southern Model Biotechnology Co., Ltd, Nanjing, China) and three C57BL/6J wild-type mice, were allocated to AS model and control groups. Mice were housed in a specific pathogen-free (SPF) facility at the Animal Experiment Center of Guizhou Medical University. ApoE^–/–^ mice were fed a high-fat diet (HFD) consisting of 40% fat and 1.25% cholesterol (Beijing Botai Honggang Biotechnology Co., Ltd., #1140B) for 8 weeks, while wild-type mice were maintained on a standard chow diet. Following anesthesia, animals were euthanized *via* intraperitoneal injection of pentobarbital sodium at a dose of 150 mg/kg. Aortic tissues were harvested post-euthanasia for subsequent analyses. All procedures complied with the animal care guidelines approved by Guizhou Medical University’s Ethics Committee (Approval Number: 2101044).

#### 2.11.2 Histology and immunostaining

Immunohistochemistry: Serial frozen sections (7 µm) of OCT-embedded aortic sinus lesions were cut at –20 °C. Sections were re-warmed, fixed, and subjected to antigen retrieval. Non-specific binding was blocked with 3% hydrogen peroxide, followed by incubation with 10% goat serum for 1 hour. Sections were then incubated overnight at 4 °C with ISG-20 primary antibody (1:200 dilution; San Ying, Wuhan, China). After washing, sections were incubated with HRP-conjugated secondary antibody (1:1000 dilution; Servicebio, Wuhan, China) for 60 minutes. Negative controls were prepared by omitting the primary antibody. Color development was performed using 3,3′-diaminobenzidine (DAB), followed by counterstaining with hematoxylin, differentiation, dehydration, and mounting with neutral resin. All immunostained images were captured using a bright-field microscope and analyzed for staining intensity using ImageJ software (NIH, USA).

Immunofluorescence Staining: Serial frozen sections (7 µm) of OCT-embedded aortic sinus lesions were cut at –20 °C. Sections were re-warmed, fixed, and subjected to antigen retrieval. After permeabilization with 0.2% Triton X-100 in PBS for 10 minutes, sections were blocked with 5% normal goat serum plus 1% BSA at room temperature for 1 hour to reduce non-specific binding. Sections were then incubated overnight at 4 °C with a primary antibody cocktail containing rat anti-mouse F4/80 (1:500; Bio-Rad Life Science Medical Products, Shanghai, China) and rabbit anti-mouse ISG20 (1:50; San Ying, Wuhan, China). After three washes with immunostaining wash buffer, slides were incubated for 1–2 hours at room temperature in the dark with a mixture of HyperFluor™ 594 goat anti-rabbit IgG (H+L) (1:500; APExBIO, Shanghai Weihuan Biotechnology, China) and FITC-conjugated goat anti-rat IgG (1:200). After additional dark washes, tissue autofluorescence was quenched with TrueBlack for 5 minutes, rinsed in PBS, and mounted with DAPI-containing anti-fade medium. Images were acquired using a laser-scanning confocal microscope with sequential scanning of DAPI (405 nm), 488 nm (ISG20), and 561 nm (F4/80) channels to eliminate cross-talk.

#### 2.11.3 Cell culture

Cell Culture and ISG20 Expression Investigation: To investigate ISG20 expression at the cellular level, RAW264.7 macrophages (STCC20020G, Servicebio, Wuhan, China) were cultured in high-glucose DMEM medium supplemented with 10% fetal bovine serum (FBS), 50 U/mL penicillin, and 50 U/mL streptomycin. When cells reached 40–50% confluency, they were stimulated with 100 μg/mL oxidized low-density lipoprotein (ox-LDL) (Solarbio, Beijing, China) for 24 hours. Post-stimulation, total protein and RNA were extracted for subsequent Western blotting and quantitative real-time polymerase chain reaction (RT-qPCR) analyses.

#### 2.11.4 Western blotting

Protein concentrations of all macrophage lysates were measured using the bicinchoninic acid (BCA) assay. Normalized protein samples were resuspended in SDS sample buffer, separated by sodium dodecyl sulfate–polyacrylamide gel electrophoresis (SDS-PAGE), and transferred onto polyvinylidene difluoride (PVDF) membranes. After blocking with 5% non-fat milk for 2 hours, membranes were incubated overnight at 4 °C with diluted primary antibodies under gentle agitation. Following washes, membranes were incubated with horseradish peroxidase (HRP)-conjugated secondary antibodies at room temperature for 1 hour. Protein bands were visualized using an enhanced chemiluminescence (ECL) system (NeoBioscience, Suzhou, China) and quantified by densitometric analysis using ImageJ software (National Institutes of Health, USA).

#### 2.11.5 Quantitative real-time polymerase chain reaction

Total RNA from the macrophages was extracted using the Total RNA Extraction Kit (NeoBioscience, Suzhou, China). For reverse transcription, 1 μg of total RNA was converted to first-strand complementary DNA (cDNA) in a 20 μL reaction volume using the Reverse Transcription Kit (Yeasen, Shanghai, China) according to the manufacturer’s instructions. SYBR Green-based RT-qPCR (Yeasen, Shanghai, China) was performed on a QuantStudio 3 Real-Time PCR System (Thermo Fisher Scientific, USA). Gene expression levels were normalized to GAPDH, and data were analyzed using GraphPad Prism 9.5 (GraphPad Software, USA). The following primers were used: Forward and reverse primers for the mouse GAPDH gene: 5′-GGTTGTCTCCTGCGACTTCA-3′ and 5′-TGGTCCAGGGTTTCTTACTCC-3′, respectively. Forward and reverse primers for the mouse ISG20 gene: 5′-TGGGCCTCAAAGGGTGAGT-3′ and 5′-CGGGTCGGATGTACTTGTCATA-3′, respectively.

## 3 Results

### 3.1 Overview of five GEO datasets

This study utilized four merged microarray datasets from the GEO database as the experimental cohort and one independent dataset for validation. The experimental cohort included 212 disease samples (atherosclerotic lesions) and 335 healthy controls, while the validation cohort comprised 16 disease samples and 13 healthy controls. For the validation cohort, gene expression values were normalized and merged using R software (version 4.3.2). Batch effects were minimized through PCA to ensure data homogeneity, thus enhancing the accuracy and reliability of subsequent analyses (**Supplementary Figure S1**).

### 3.2 Identification of differentially expressed genes

**Figure 1:**
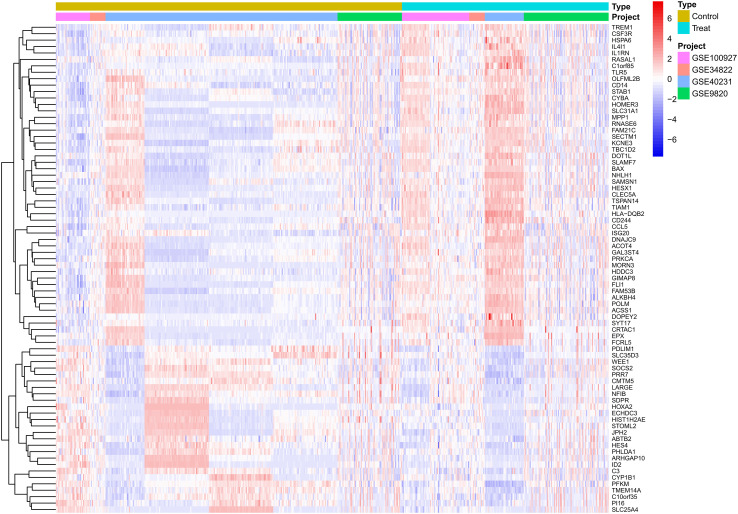
Heatmap of differentially expressed genes (DEGs) in atherosclerotic plaque versus healthy arterial tissues. The heatmap displays the expression patterns of significantly differentially expressed genes identified from the analysis of the adjusted GSE dataset. Each row represents a single gene, and each column represents an individual tissue sample. Samples are grouped into two primary clusters: healthy arterial tissue and atherosclerotic plaque tissue. The color scale illustrates the relative gene expression levels, where red denotes up-regulation and blue denotes down-regulation in atherosclerotic plaques compared to healthy controls. Hierarchical clustering reveals co-expression patterns among genes across the different tissue types. DEGs, Differentially expressed genes; GSE, Gene Expression Omnibus Series.

**Figure 2:**
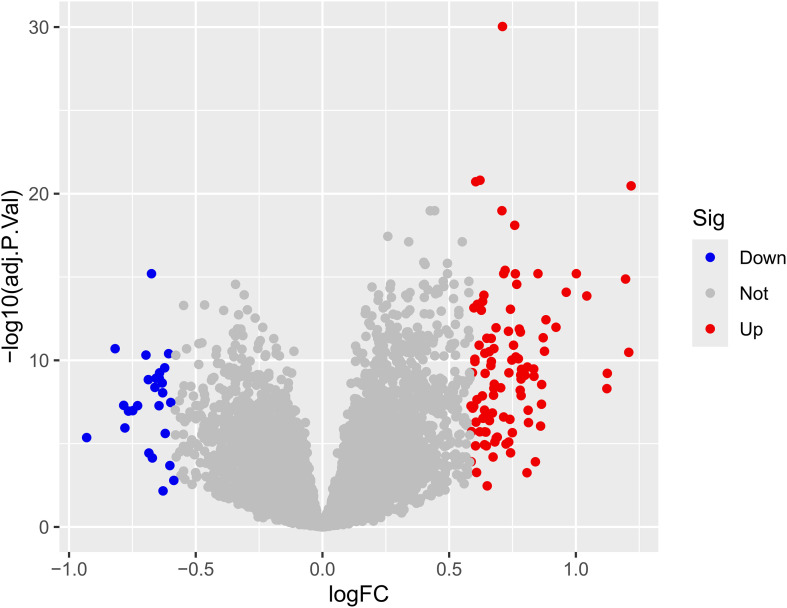
Volcano plot of differential expression analysis. The volcano plot displays the relationship between the logarithmic fold change (logFC) and the statistical significance (-log_10_ adjusted p-value) for each feature. Points are colored based on their differential expression status: Down (significantly decreased), Not (not significant), and Up (significantly increased). The horizontal dashed line indicates the threshold for statistical significance (typically adjusted p-value < 0.05), while vertical dashed lines represent the logFC thresholds (e.g., ± 0.5 or ±1.0). logFC, Logarithm of fold change, indicating the magnitude of expression difference; adj. P.Val, Adjusted p-value, corrected for multiple testing; Sig, Significance category based on defined thresholds for logFC and adjusted p-value.

The results indicated that smaller P-values were associated with greater reliability in gene ranking and differential expression. A total of 98 upregulated and 25 downregulated DEGs were identified (**Supplementary Table S1** for detailed gene annotations). A heatmap (**Figure 1**) visualized the expression patterns of the top 50 upregulated DEGs and all 25 downregulated DEGs, showing clear clustering between atherosclerotic and control samples. The volcano plot (**Figure 2**) further illustrated genome-wide differential expression across the integrated GEO datasets, highlighting genes surpassing the significance threshold (adjusted P < 0.05, logFC > 0.585).

### 3.3 Mendelian randomization analysis

**Figure 3:**
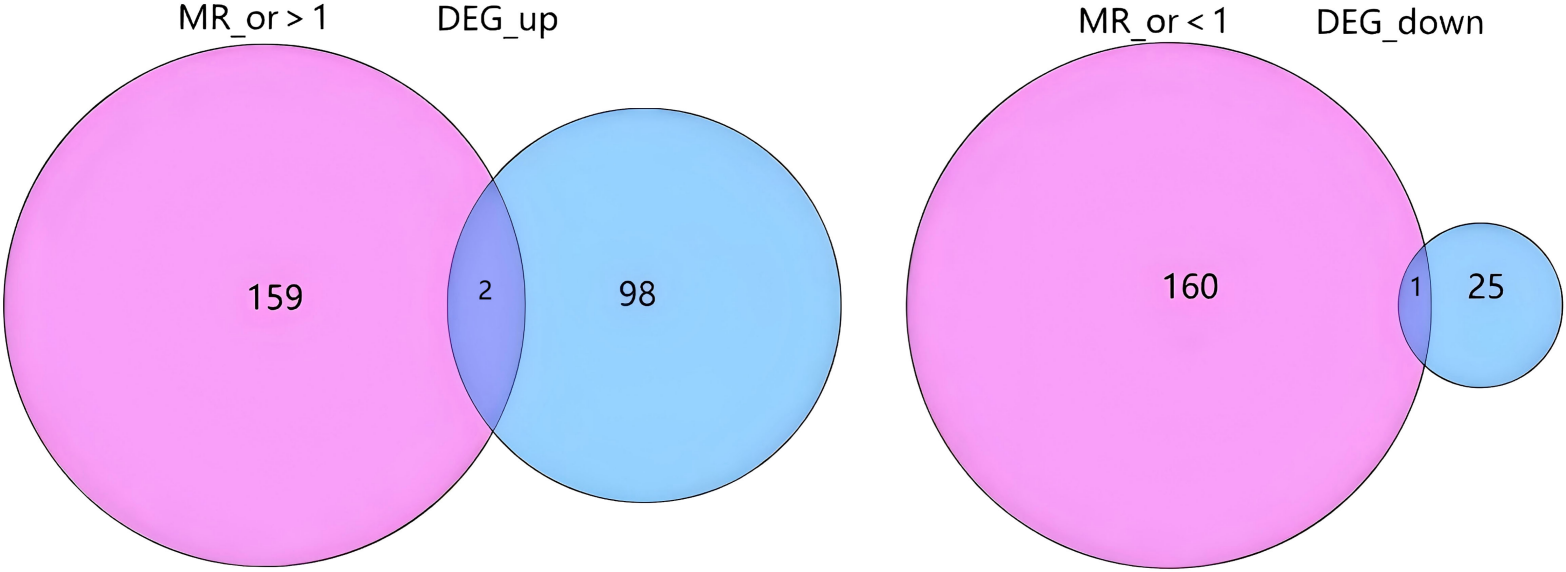
Overlap between Mendelian randomization results and differential gene expression. The diagram illustrates the intersection between genes with a significant causal effect from Mendelian randomization analysis and differentially expressed genes (DEGs). The left side shows the overlap between genes with an MR odds ratio (MR_or) > 1 (indicating risk effects) and up-regulated DEGs (DEG_up). The right side shows the overlap between genes with an MR_or < 1 (indicating protective effects) and down-regulated DEGs (DEG_down). The numbers represent the count of genes in each segment. MR_or, Mendelian randomization odds ratio; DEG, Differentially expressed gene; DEG_up, Up-regulated differentially expressed genes; DEG_down, Down-regulated differentially expressed genes.

following stringent filtering, 25,224 SNPs were retained as IVs, all of which met the core assumptions of MR. All selected SNPs exhibited F-statistics > 10 (**Supplementary Table S2** for detailed SNP characteristics). Using MR analysis and five predefined filtering criteria, 319 AS-associated genes were identified (**Supplementary Table S3**). Cross-analysis revealed three overlapping genes between disease-related genes and MR-derived eQTL results: two upregulated genes (CLEC5A, ISG20) and one downregulated gene (HOXA2) (**Figure 3**).

Subsequent MR analysis of these key genes demonstrated their causal effects on AS. IVW analysis revealed significant positive causal associations for the upregulated genes: CLEC5A (OR = 1.001, 95% CI: 1.001–1.005, P = 0.047) and ISG20 (OR = 1.001, 95% CI: 1.002–1.007, P = 0.030). In contrast, the downregulated gene showed a significant negative causal association: HOXA2 (OR = 0.997, 95% CI: 0.997–0.998, P = 0.016). Consistently, all MR methods confirmed increased disease risk for upregulated genes (OR > 1) and reduced risk for the downregulated gene (OR < 1) (**Table 1**). Heterogeneity (Cochran’s Q test, P > 0.05) and horizontal pleiotropy (MR-Egger intercept test, P > 0.05) analyses revealed no significant bias, indicating negligible confounding effects. Sensitivity analysis *via* the leave-one-out method demonstrated that excluding individual SNPs did not significantly alter effect estimates, supporting the robustness of the causal inferences (**Supplementary Table S4** for full results).

**Table 1:** The table shows the causal relationship between three key genes and atherosclerosis.

Exposure	nsnp	Method	OR (95% CI)	P value
CLEG5A	9	Weighted median	1.001 (1.000 to 1.003)	0.133
		Inverse variance weighted	1.002 (1.000 to 1.003)	0.048
HOXA2	3	Weighted median	0.998 (0.996 to 1.000)	0.019
		Inverse variance weighted	0.998 (0.996 to 1.000)	0.016
ISG20	7	Weighted median	1.001 (0.999 to 1.003)	0.301
		Inverse variance weighted	1.002 (1.000 to 1.003)	0.030

SNP, single nucleotide polymorphism; nsnp, number of instrumental single nucleotide polymorphisms; OR, odds ratio; CI, confidence interval; CLEG5A, C-type lectin domain family 5 member A; HOXA2, homeobox A2; ISG20, interferon-stimulated exonuclease gene 20.

The Weighted median and Inverse variance weighted methods are two distinct analytical methods used in Mendelian randomization analysis.

**Figure 4:**
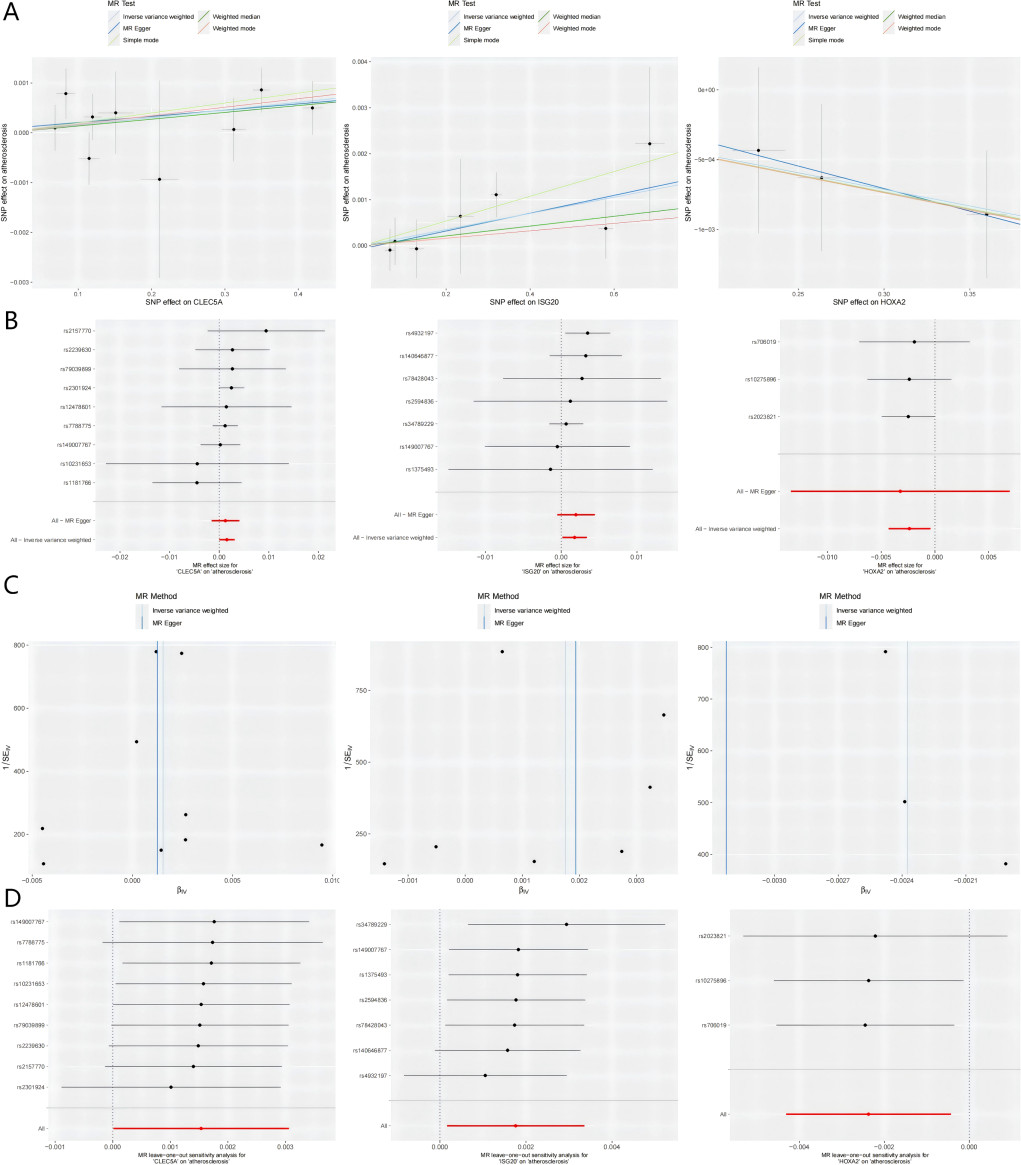
Integrated visualization of three key genes through. **(A)** Scatter Plots: Illustrates the relationship between the SNP-exposure association (x-axis) and the SNP-outcome association (y-axis). The slope of each fitted line represents the causal estimate from the corresponding MR method. **(B)** Forest Plots: Displays the causal estimate (with 95% confidence interval) for each individual instrumental SNP and the combined MR methods. **(C)** Funnel Plots: Assesses potential directional pleiotropy. Rough symmetry suggests the absence of major pleiotropic bias. **(D)** Leave-one-out Plots: Demonstrates the influence of each individual SNP on the overall IVW causal estimate by iteratively removing one SNP at a time. MR, Mendelian randomization; SNP, single nucleotide polymorphism; CLEC5A, C-type lectin domain containing 5A; HOXA2, Homeobox A2; ISG20, Interferon stimulated exonuclease gene 20; IVW, Inverse variance weighted; SE, Standard error.

Visual summaries of the three prioritized genes—including scatter plots (exposure-outcome linearity), forest plots (effect size consistency), funnel plots (instrument symmetry), and leave-one-out sensitivity analyses—are presented in **Figure 4**. The chromosomal locations of these genes are annotated on a circular GO map (**Supplementary Figure S2**), illustrating their functional and positional relevance within the genome.

### 3.4 Functional annotation

The functional implications of the three key genes were further investigated. GO analysis revealed their primary involvement in biological processes such as osteoblast development and differentiation, viral assembly and lifecycle regulation, and negative regulation of myeloid cell apoptosis (**Supplementary Figures S3A–C**).

Pathway activity analysis, based on the expression levels of the three key genes, revealed distinct immunobiological divergences. In the high-expression group of CLEC5A, associated pathways included immunoregulatory processes such as chemokine signaling, cytokine-receptor interaction, response to Leishmania infection, lysosomal activity, and Toll-like receptor signaling (**Supplementary Figure S4**). Conversely, the low-expression group was linked to genome maintenance mechanisms, including base excision repair, mismatch repair, nucleotide metabolism (purine/pyrimidine), and ribosome biogenesis. ISG20 exhibited a clear functional dichotomy: its high-expression state was associated with cell cycle regulation, recurrent cytokine-receptor interactions, JAK-STAT signaling, and T-cell receptor activation, while the low-expression profile indicated metabolic reprogramming, including lysosomal function, glycosaminoglycan catabolism, oxidative phosphorylation, and the coordinated degradation of porphyrin/chlorophyll metabolites and branched-chain amino acids (valine/leucine/isoleucine) (**Supplementary Figure S5**). Notably, the high-expression group of HOXA2 was linked to distinct immunopathological processes, such as those involved in asthma pathogenesis, complement and coagulation cascades, extracellular matrix (ECM)-receptor interaction, and systemic lupus erythematosus markers (**Supplementary Figure S6**). In contrast, the low-expression group showed no significant pathway activity.

Functional annotation of the three key genes revealed distinct immunobiological signatures linked to their expression levels. For CLEC5A, high expression was associated with innate immune activation, including cellular responses to biotic stimuli, granulocyte migration—particularly neutrophil trafficking—modulation of TNF superfamily signaling, and enhanced immune activation. Conversely, low expression was linked to ribosomal organization and structural assembly. ISG20 demonstrated a marked functional dichotomy, with high expression driving adaptive immune processes such as leukocyte adhesion and T-cell activation, while low expression correlated with metabolic reprogramming involving lipid catabolism, monocarboxylic acid metabolism, ficolin-1-rich granule formation, and vacuolar membrane reorganization. HOXA2 exhibited unique associations with immunomodulatory and developmental processes: its high expression was linked to extracellular matrix remodeling *via* cell adhesion complexes and integrin binding, as well as primitive germ layer specification. Low expression, on the other hand, was associated with specialized transport processes, including aromatic compound export and copper ion detoxification, along with enhanced regulation of reactive oxygen species biosynthesis (**Supplementary Figures S7A–C**).

Integrated analysis revealed that these three key genes contribute to atherogenesis through coordinated regulation of immune and metabolic processes. The high-expression state of CLEC5A was primarily characterized by activation of pro-inflammatory immune pathways, including chemokine signaling, cytokine-receptor interactions, and Toll-like receptor signaling, suggesting its central role in vascular inflammatory responses. ISG20 exhibited the most pronounced functional dichotomy: its high-expression profile was defined by T-cell receptor signaling, JAK-STAT activation, and cytokine interactions, indicating direct involvement in immune-inflammatory activation during atherosclerotic plaque progression. Conversely, its low-expression state showed features of metabolic reprogramming, such as oxidative phosphorylation, branched-chain amino acid metabolism, and lysosomal activity, suggesting a potential role in modulating foam cell formation and plaque stability through metabolic and clearance mechanisms. HOXA2 displayed specific associations with extracellular matrix interactions and the complement and coagulation cascade, indicating a potential role in vascular remodeling and thrombotic processes. Collectively, these findings systematically elucidate how these three genes participate in atherosclerotic pathogenesis through distinct yet complementary immunometabolic mechanisms.

### 3.5 Immune cell infiltration analysis

**Figure 5:**
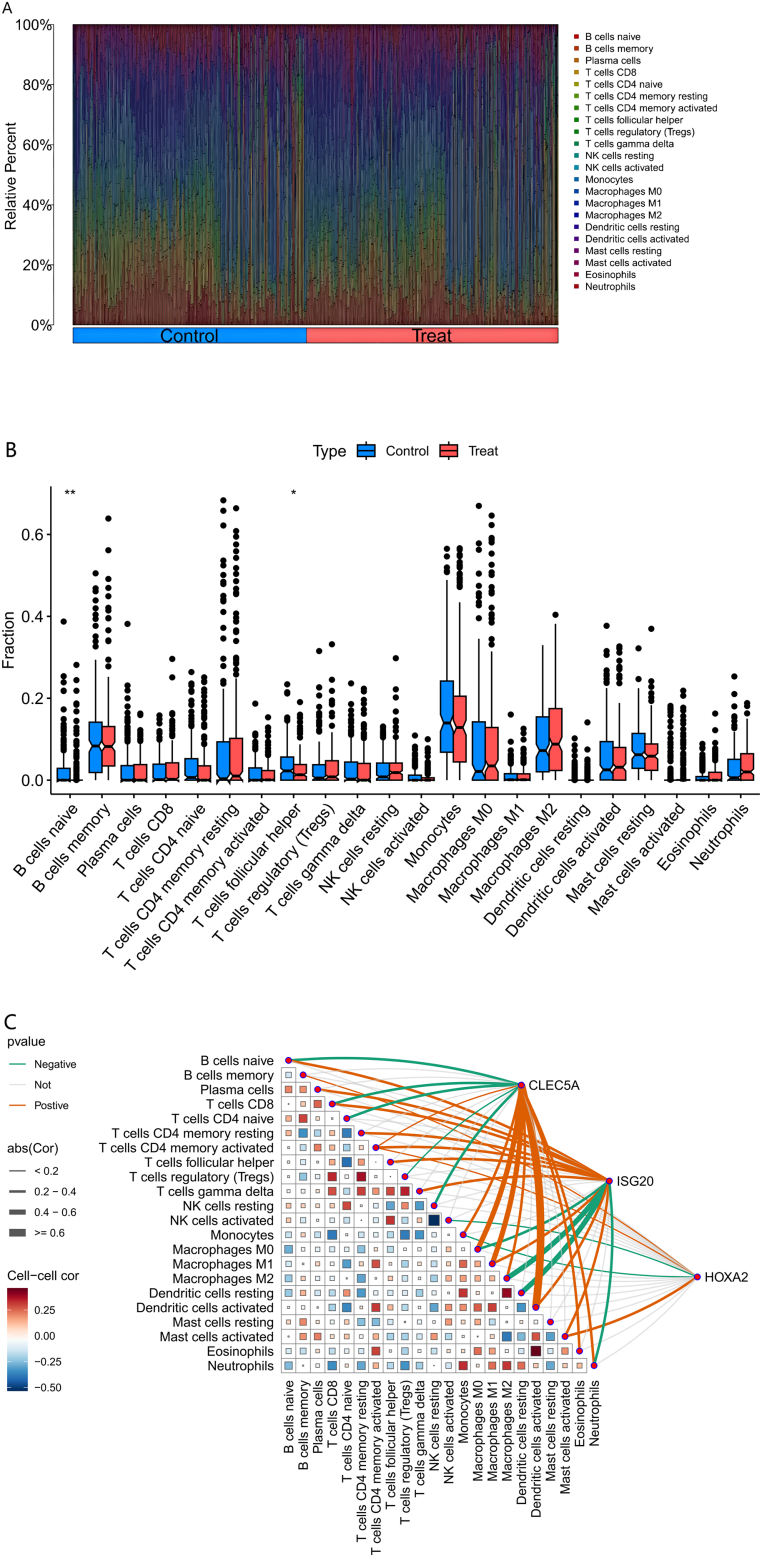
Immune Cell Infiltration Analysis. (Figure **(A)** Comparative composition of immune cell infiltration in atherosclerotic plaques.) The bar chart delineates the relative proportions of 22 immune cell subtypes between healthy (control) and pathological (treatment) conditions. Each bar represents the complete immune cell repertoire of an individual sample, with cumulative proportions normalized to 100%. (Figure **(B)** Differential immune cell infiltration associated with plaque vulnerability.) The graph delineates specific disparities in immune cell abundance between healthy (control) and diseased (treatment) conditions, with asterisks denoting statistical significance of the observed differences. (Figure **(C)** Correlation between MR-prioritized causal genes and immune contexture in atherosclerosis.) The heatmap depicts the Spearman correlation coefficients between the expression of causal genes (CLEC5A, HOXA2, ISG20) and the infiltration levels of various immune cells in atherosclerotic plaques. Immune cell fractions were estimated from bulk transcriptome data using the CIBERSORT deconvolution algorithm. Statistical significance is denoted as follows: *p < 0.05, **p < 0.01, ***p < 0.001. Tregs, regulatory T cells; NK cells, natural killer cells; Cor, Correlation coefficient.

The CIBERSORT deconvolution algorithm was systematically employed to quantify immune cell infiltration landscapes and examine the relationships between co-expressed genes and immunophenotypic profiles in AS. Immune profiling across all samples revealed heterogeneous distributions of 22 immune cell subtypes, with notable depletion of naïve B cells (CD19+CD27−IgD+) and follicular helper T cells (CD4+CXCR5+PD-1+) in atherosclerotic (AS) lesions compared to control tissues (P < 0.01, **Figures 5A, B**). Correlation analysis with the 22 immune cell types (**Figure 5C**) revealed distinct association patterns for the co-expressed genes. Specifically, CLEC5A showed significant positive correlations with pro-inflammatory immune cells, including strong positive correlations with M1 macrophages (Cor ≥ 0.4, p < 0.05), activated neutrophils (Cor ≥ 0.4), and activated CD8+ T cells (Cor = 0.25), highlighting its critical role in inflammatory responses. Additionally, it exhibited significant negative correlations with regulatory T cells (Tregs, Cor = -0.5, p < 0.05) and M2 macrophages (Cor = -0.25), suggesting its potential to promote inflammatory progression by suppressing immunosuppressive microenvironments. ISG20 demonstrated broad associations with adaptive immune cells, showing moderate-to-strong positive correlations (p < 0.05) with activated dendritic cells (Cor = 0.4–0.6), activated NK cells (Cor = 0.25–0.4), and activated CD4+ memory T cell subsets (Cor = 0.2–0.4), indicating its involvement in antigen presentation and antiviral immune regulation. In contrast, weak negative correlations were observed with resting mast cells (Cor = -0.25) and M0 macrophages (Cor = -0.2), implying potential regulation by cellular activation states. HOXA2 exhibited a more heterogeneous anti-inflammatory signature, positively correlating with eosinophils (Cor ≥ 0.4), resting dendritic cells (Cor = 0.25–0.4), and M2 macrophages (Cor = 0.2–0.4, p < 0.05), while inversely associating with activated CD4+ memory T cells (Cor = -0.25) and neutrophils (Cor = -0.2), suggesting its role in maintaining immune homeostasis by balancing pro- and anti-inflammatory cell subsets. Collectively, these findings emphasize that CLEC5A, ISG20, and HOXA2 regulate immune dynamics through divergent mechanisms—pro-inflammatory activation, adaptive immune modulation, and anti-inflammatory equilibrium, respectively—providing molecular insights into immune crosstalk within pathological microenvironments.

### 3.6 Validation cohort differential analysis

**Figure 6:**
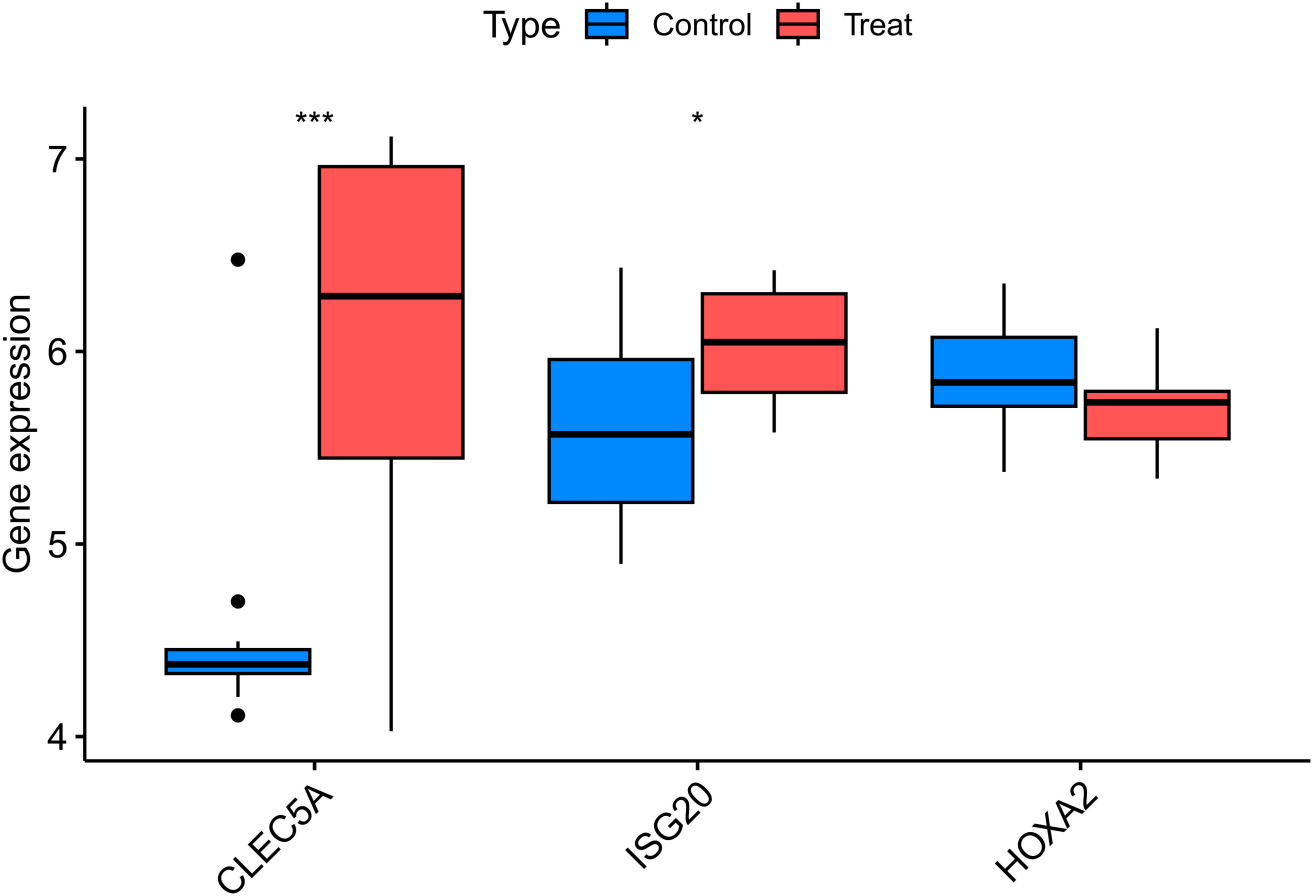
Validation of key differentially expressed genes in the independent cohort GSE28829. Box plots comparing the expression levels of three key genes (CLEC5A, ISG20, and HOXA2) between atherosclerotic plaque tissues and healthy arterial tissues from the GEO dataset GSE28829. The expression patterns of these genes were consistent with the discovery cohort, confirming their roles in atherosclerosis. The center line in each box represents the median, the bounds of the box indicate the interquartile range (IQR), and the whiskers extend to the minimum and maximum values within 1.5×IQR. CLEC5A,C-type Lectin Domain Family 5 Member A; ISG20,nterferon Stimulated Exonuclease Gene 20kDa; HOXA2,Homeobox A2; Control, Healthy Arterial Tissue; Treat, Atherosclerotic Plaque Tissue. *p < 0.05, ***p < 0.001.

Following data annotation and quality control of dataset GSE28829 (validation cohort), the expression levels of the three key genes were validated. The results showed that the upregulated genes CLEC5A and ISG20 exhibited significantly higher expression in the experimental group (AS samples) compared to healthy controls (P < 0.01 and P < 0.05, respectively). In contrast, the downregulated gene HOXA2 showed no significant differential expression between the experimental and control groups (**Figure 6**).

### 3.7 Experimental validation

**Figure 7:**
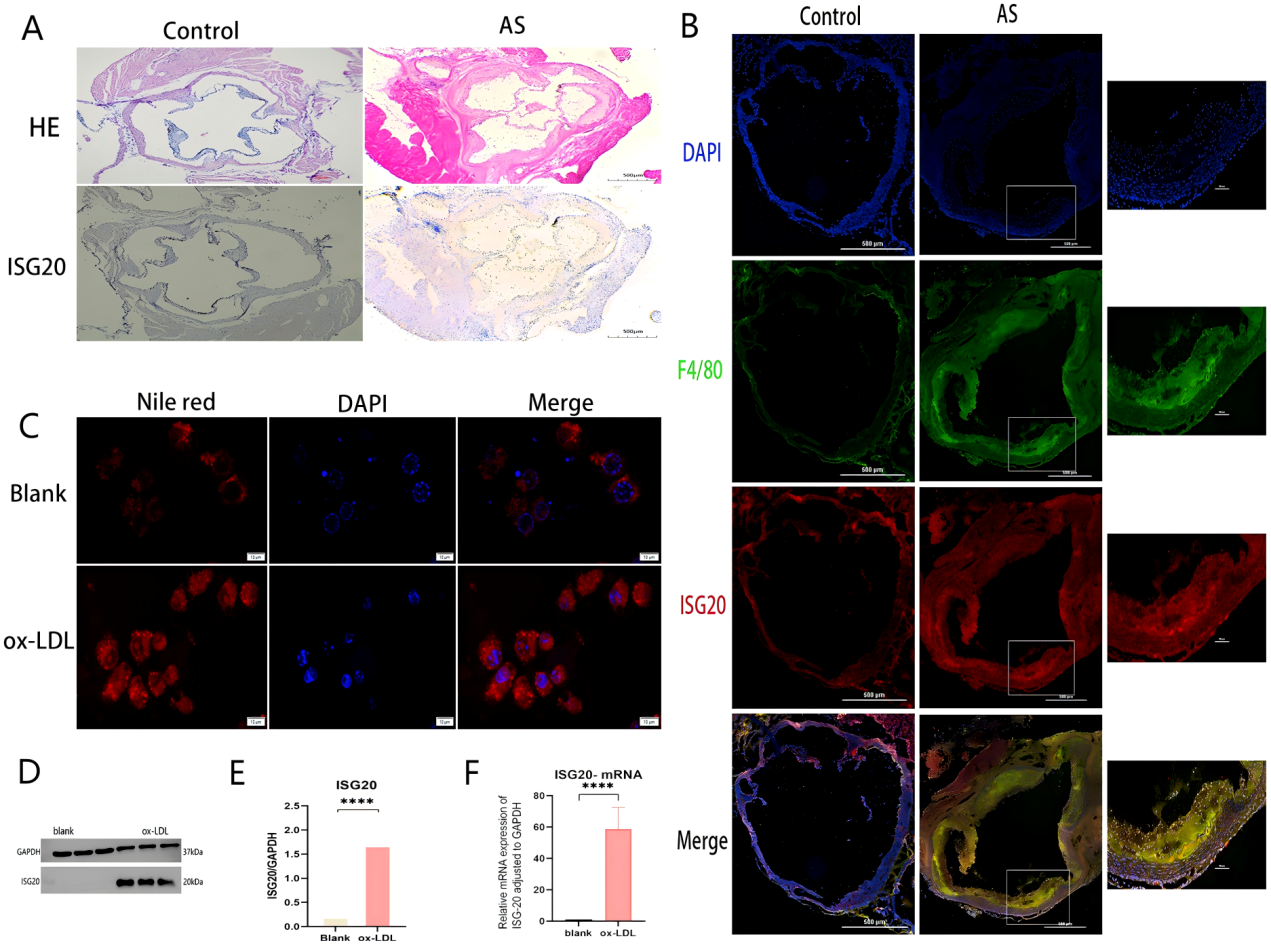
ISG20 expression is upregulated in atherosclerotic plaques and induced by ox-LDL in macrophages. **(A)** Histological analysis of the aortic root from ApoE^–/–^ mice fed a high-fat diet (HFD). Hematoxylin and eosin (H&E) staining shows a typical advanced atherosclerotic plaque. The lesion area exhibits massive foam cell accumulation, an extracellular lipid core, and disrupted cellular architecture. Immunohistochemical staining of an adjacent section reveals strong nuclear ISG20-positive signals within the atherosclerotic lesion, compared to the adjacent normal vessel area. **(B)** Immunofluorescence co-staining of aortic root sections for ISG20 (red) and the macrophage marker F4/80 (green). Nuclei are counterstained with DAPI (blue). The merged image shows extensive co-localization (yellow) of ISG20 with F4/80-positive macrophages infiltrating the plaque. **(C)** Representative images of Nile Red staining in RAW264.7 macrophages treated with or without 100 µg/mL ox-LDL for 24 hours. Bright red fluorescence indicates intracellular lipid droplets. Ox-LDL treatment induced robust foam cell formation. **(D)** Western blot analysis of ISG20 protein expression in cells treated with vehicle (control) or ox-LDL. GAPDH served as a loading control. **(E)** Quantitative analysis of ISG20 protein expression normalized to GAPDH in control versus ox-LDL-treated cells. Data are presented as mean ± SEM; P < 0.01 (Student’s t-test). **(F)** RT-qPCR analysis of ISG20 mRNA expression in control versus ox-LDL-treated cells, normalized to GAPDH. Data are presented as mean ± SEM; P < 0.0001. Scale bar: 500 μm, Statistical significance was determined by Student’s t-test, ***p < 0.0001, ****p < 0.0001. DAPI, 4’,6-diamidino-2-phenylindole; F4/80, A specific surface marker for murine macrophages; ox-LDL, oxidized low-density lipoprotein; qRT-PCR, quantitative reverse transcription polymerase chain reaction; HE, hematoxylin and eosin staining; AS, atherosclerosis; SD, standard deviation.

To investigate the role of ISG20 in the development and progression of AS, its expression was examined in an atherosclerotic model. The aortas of ApoE^–/–^ mice fed an HFD displayed clear features of AS, and histological analysis was performed on sections of the aortic root. As shown in **Figure 7A**, H&E staining of the aortic root from HFD-fed ApoE^–/–^ mice revealed advanced atherosclerotic plaques. These lesions showed significant foam cell accumulation, formation of an extracellular lipid core, and disruption of the internal cellular architecture, confirming successful establishment of the atherosclerotic model. Immunohistochemical staining for ISG20 on adjacent sections revealed substantial changes. Compared to surrounding normal vascular tissue, intense ISG20-positive signals were detected within the atherosclerotic lesions. These signals were predominantly localized in the nuclei of cells in the plaque core, suggesting marked activation of intracellular ISG20 expression in lesioned areas. In summary, the results in **Figure 7A** indicate that ISG20 is specifically highly expressed in the atherosclerotic plaques of the aortic root in the HFD-induced ApoE^–/–^ mouse model, highlighting its potential involvement in the pathological process of AS.

To further identify the cellular source of ISG20 within atherosclerotic lesions, immunofluorescence co-staining was performed on aortic root sections from HFD-fed ApoE^–/–^ mice. A specific antibody against ISG20 (red) and an antibody against the macrophage marker F4/80 (green) were used, with DAPI (blue) for nuclear counterstaining. As shown in **Figure 7B**, numerous F4/80-positive macrophages (green signals) were observed infiltrating the atherosclerotic plaque regions. More importantly, ISG20 signals (red) were widely present within the plaques and showed considerable overlap with F4/80-positive macrophage areas. The merged channels revealed abundant yellow fluorescent signals (indicating co-localization of red and green) in macrophage-enriched regions, demonstrating specific ISG20 expression in plaque macrophages. Notably, not all DAPI-positive nuclei exhibited ISG20 signals, indicating cell-specific expression. This co-localization analysis strongly suggests that macrophages infiltrating the lesion sites serve as a significant cellular source of ISG20 during AS progression.

To investigate the direct effect of ox-LDL, a key pathogenic factor in AS, on ISG20 expression, RAW264.7 cells were treated with 100 μg/mL ox-LDL for 24 hours to induce foam cell formation. As shown in **Figure 7C**, Nile Red staining revealed numerous bright red lipid droplets within the cells of the ox-LDL-treated group, which were significantly increased compared to the control group, confirming successful induction of the foam cell model. To quantitatively assess the regulatory effect of ox-LDL on ISG20 at the protein level, ISG20 protein expression was evaluated in macrophages treated with or without ox-LDL (100 μg/mL, 24 hours) using Western blot analysis, with GAPDH as a loading control. A representative Western blot image (**Figure 7D**) clearly showed a significantly intensified ISG20 protein band signal in ox-LDL-stimulated macrophages compared to the untreated control. Quantitative densitometric analysis of three independent experiments was performed, and statistical analysis of protein levels normalized to GAPDH (mean ± SD) confirmed that ox-LDL treatment significantly increased ISG20 protein levels in macrophages, with a statistically significant difference compared to the control group (***p < 0.001, **Figure 7E**). To examine whether ox-LDL regulation of ISG20 expression occurs at the transcriptional level, total RNA was extracted from macrophages treated with or without ox-LDL (50 μg/mL, 24 hours), and ISG20 mRNA expression levels were analyzed by quantitative real-time PCR (qPCR). Gene expression was normalized using GAPDH as the internal reference gene. As shown in **Figure 7F**, qPCR analysis revealed that ox-LDL treatment significantly elevated ISG20 mRNA expression in macrophages compared to the control group, with a statistically significant difference (mean ± SD, n = 3 independent experiments, ***p < 0.001). These results were consistent with the findings at the protein level (**Figures 7D, E**), collectively confirming that ox-LDL significantly activates ISG20 expression in macrophages, starting at the transcriptional level. This suggests that ox-LDL may directly participate in the transcriptional regulation of the ISG20 gene, potentially through transcription factors or related signaling pathways, thereby promoting sustained high expression of ISG20 in atherosclerotic lesions.

## 4 Discussion

AS, a chronic inflammatory vascular disease, involves complex etiological mechanisms. A key factor in its progression is the accumulation of macrophages, which transform into foam cells and persist within the vascular wall (3). Therefore, identifying macrophage-related biomarkers is crucial for advancing strategies to prevent CVD. In this study, integrated analyses of multiple datasets from the GEO database were conducted to identify DEGs. By combining GWAS and eQTL data with MR analysis, AS-associated genes, including CLEC5A, ISG20, and HOXA2, were systematically screened. Functional enrichment and pathway analyses were further performed to explore the mechanisms and pathways linked to these key genes. Notably, CLEC5A and ISG20 exhibited significant causal relationships with AS, emerging as risk genes contributing to disease pathogenesis. In contrast, HOXA2 showed no statistically significant association in our analyses. Additionally, ISG20 was validated through cellular experiments and plaque assays in APOE−/− mouse models. The validation results confirmed that ISG20 is highly expressed in foam macrophages and significantly upregulated within atherosclerotic plaques, a process directly induced by the key atherogenic factor ox-LDL. These findings provide novel insights into the pathogenesis of AS and position ISG20 as a potential therapeutic target for its intervention.

Our MR analysis revealed a causal relationship between the CLEC5A gene and AS, indicating that elevated expression of CLEC5A is associated with an increased risk of disease onset. Validation cohort results further confirmed the consistency of these findings. CLEC5A (C-type lectin domain family 5 member A), a member of the C-type lectin superfamily, is a type II transmembrane protein primarily expressed in myeloid cells, including macrophages, monocytes, neutrophils, and dendritic cells (11). CLEC5A binds to the adaptor protein DNAX-activating protein 12 (DAP12), leading to the activation of spleen tyrosine kinase (SYK), which subsequently triggers downstream signaling pathways. This cascade induces the massive release of cytokines and inflammatory mediators, such as interleukin-6 (IL-6), tumor necrosis factor (TNF), and interleukin-8 (IL-8), from host cells. This process is critical for innate immune responses and effective defense against pathogen invasion (12). As a pattern recognition receptor (PRR) for viruses and bacteria, CLEC5A recognizes diverse pathogen-associated molecular patterns (PAMPs) and damage-associated molecular patterns (DAMPs), thereby activating immune cells and initiating immune responses. In flavivirus infections (e.g., dengue and Japanese encephalitis viruses), CLEC5A serves as a key receptor that drives the production of high levels of pro-inflammatory cytokines and chemokines (13, 14). Additionally, CLEC5A plays a vital role in combating bacterial infections, with studies showing its critical function in Listeria monocytogenes infection, promoting IL-1β production, and recognizing other bacteria such as Staphylococcus aureus and Klebsiella pneumoniae, contributing to host immune defense (15).

CLEC5A, predominantly expressed on macrophages and neutrophils, induces inflammatory responses upon activation. In this study, MR analysis combined with machine learning identified a significant causal relationship between CLEC5A and AS, with elevated CLEC5A expression associated with an increased risk of the disease. Furthermore, CLEC5A expression was positively correlated with immune cells, such as macrophages and activated macrophages. Previous studies in AS models demonstrated that MDL-1 (CLEC5A) expression in macrophages within lesion areas is closely linked to inflammatory responses. Specifically, in regression models of AS, significant downregulation of MDL-1 expression was observed in macrophages from regressive plaques of LDLR−/− mice, accompanied by reduced macrophage infiltration and decreased pro-inflammatory M1 macrophage markers (16). These animal studies corroborate our findings, suggesting that MDL-1 may serve as a biomarker for monitoring early atherosclerotic plaque progression. Additionally, CLEC5A is highly expressed in inflammatory M1-polarized myeloid cells but shows moderate expression in tumor-associated phenotypes (M2c or TAM) (17). This suggests that the association between CLEC5A and AS is primarily driven by its activation in macrophages within atherosclerotic lesions, particularly in pro-inflammatory M1 macrophages, which may contribute to disease progression and inflammation. AS, as a complex inflammatory disorder, involves the recruitment and proliferation of diverse immune cells. These cells interact and release inflammatory mediators, creating a vicious cycle that promotes plaque formation and destabilization (18). CLEC5A, as a receptor expressed on immune cells, participates in diverse immune responses by activating multiple signaling pathways and promoting the release of chemokines, thereby regulating immune cell migration and proliferation (19). These findings collectively suggest that CLEC5A plays a critical role in the progression of AS. However, the precise molecular mechanisms underlying CLEC5A’s function in AS remain incompletely understood. Further studies are warranted to explore its clinical implications and therapeutic potential as a novel target.

ISG20, a key protein in host antiviral innate immunity, plays diverse functional roles, with its expression regulated by interferons. Specifically, type I (α/β) and type II (γ) interferons can induce ISG20 through the interferon-stimulated response element (ISRE) in its promoter region, thereby promoting innate immune responses (20). In the present study, a series of *in vivo* and *in vitro* experiments systematically revealed the expression and regulatory mechanisms of ISG20 in AS. This study initially found that ISG20 protein is specifically highly expressed in the aortic root plaques of HFD-fed ApoE^–/–^ mice (**Figure 7A**). Further immunofluorescence co-localization analysis precisely localized ISG20 expression within the plaques to infiltrating macrophages (**Figure 7B**). Under pro-atherogenic stimulation, ox-LDL—a key pathogenic factor in AS—directly and significantly upregulates both mRNA and protein expression of ISG20 in RAW264.7 macrophages *in vitro* (**Figures 7C–F**). This coherent experimental evidence not only validates our bioinformatic predictions but also dissociates ISG20 induction from its conventional interferon-dependent context, repositioning it within a signaling environment dominated by metabolic stress and lipid dysregulation. Based on these integrated results, this study proposes a potential working model: in the atherosclerotic microenvironment, transcription of the ISG20 gene in intimal-infiltrated macrophages is significantly activated under the persistent stimulation of ox-LDL, leading to substantial accumulation of ISG20 protein within the nucleus.

AS is fundamentally a chronic inflammatory disease, with macrophages serving as central players in the inflammatory response (21). Given that ISG20 is a nuclear protein with 3’→5’ RNA exonuclease activity, its functions are typically associated with RNA metabolism, viral defense, and cellular stress responses (22). Thus, ISG20 may represent a critical node within the ox-LDL-triggered, macrophage-mediated circuit of amplified inflammation and homeostatic imbalance. As a nuclear exonuclease, the most plausible functional impact of ISG20 in atherogenesis is the regulation of specific mRNA stability. One potential pro-inflammatory mechanism is that ISG20 degrades repressive mRNAs, thereby releasing a “brake” on inflammatory signaling. Previous research suggests that ISG20 may activate inflammatory cascades *via* its RNA-binding and exonuclease activities. For instance, ISG20 has been shown to recognize aberrant RNA species (e.g., dsRNA or oxidized RNA) and trigger downstream signaling pathways. By analogy, it is hypothesized that in the ox-LDL-induced stress environment, ISG20 might similarly sense oxidized nucleic acids (such as mitochondrial DNA or oxidized RNA), thereby promoting sustained activation of pro-inflammatory signaling pathways like the NLRP3 inflammasome and NF-κB, exacerbating plaque inflammation (22). This aligns with the strong ISG20 signal observed, which correlates with lesion severity (**Figure 7A**). Furthermore, while ISG20 has been shown to degrade oxidized RNA species to mitigate cellular damage (23), its elevated expression in the context of ox-LDL exposure might paradoxically amplify endoplasmic reticulum (ER) stress and trigger apoptotic signaling, affecting cell survival in the plaque core and thereby promoting necrotic core formation—a key determinant of plaque instability (24). This corresponds well with our observation of ox-LDL-induced high ISG20 expression accompanied by substantial lipid droplet accumulation (**Figure 7C**). Collectively, these mechanisms position ISG20 as a molecular accelerator exacerbating AS through multiple axes: inflammation, lipid metabolism, and cell death.

Although this study experimentally validated the expression profile and upstream regulation of ISG20 as a ‘risk gene’, several limitations remain. Firstly, our functional evidence is not yet complete, with the most critical next step being the elucidation of the precise biological function of ISG20. Utilizing macrophage-specific ISG20 knockout ApoE^–/–^ mouse models would provide the gold standard for definitively establishing its role as a ‘driver’ rather than a ‘bystander’ *in vivo*. ISG20 deficiency would alleviate plaque burden, reduce inflammatory infiltration, and enhance plaque stability. Secondly, our understanding of its downstream mechanisms is still at the hypothesis stage. Future studies should integrate RNA immunoprecipitation sequencing (RIP-seq) with high-throughput transcriptomics (RNA-seq) to directly identify the target RNAs bound by ISG20 in macrophages and delineate its impact on the global gene expression network, uncovering the molecular basis of its pro-atherogenic role at the ‘target’ level. While this study focused on macrophages, the potential roles of ISG20 in other cell types (e.g., endothelial cells, smooth muscle cells) should also be explored. Validation of our findings in human atherosclerotic plaque samples would further strengthen their clinical relevance.

Current research on the direct relationship between HOXA2 and AS is limited. In our analysis, no significant association was detected between this gene and AS. However, validation cohort analysis revealed a declining trend in disease risk with elevated HOXA2 expression. Notably, studies have identified altered methylation levels of specific genes (HOXA2, HOXD4, and the imprinted gene MEST) in atherosclerotic plaques compared to normal tissues, characterized by a hypomethylation state (25). The Hox gene family plays a pivotal role in embryonic development, including the formation of the hindbrain, neural crest cells, and brain tissues in both mice and humans (26). HOXA2 is critically involved in cell differentiation and migration, where it suppresses chondrogenic differentiation during cartilage formation and regulates cellular migration. Additionally, HOXA2 inhibits oligodendrocyte differentiation while promoting their proliferation (27). Members of the HOXA2 gene family have also been implicated in the development of various cancers (28–30). Under normal physiological conditions, DNA methylation regulates gene expression and maintains genomic stability, whereas hypomethylation may lead to aberrant gene activation. This suggests that the methylation status of the HOXA2 gene may play a significant role in AS development, although the precise mechanisms and influencing factors require further investigation. Furthermore, studies on the homologous gene HOXA-AS2 reveal that the long non-coding RNA (lncRNA) HOXA-AS2 acts as a critical suppressor of endothelial inflammation and is significantly associated with carotid AS. In human peripheral blood monocytes, HOXA-AS2 inhibits NF-κB signaling pathway activation by controlling IκBα degradation and Rela acetylation at the K310 site, thereby suppressing inflammatory responses. Notably, NF-κB reciprocally activates the transcriptional elongation of HOXA-AS2, forming a negative feedback loop. These findings suggest that HOXA-AS2 may serve as a key therapeutic target for vascular diseases linked to endothelial inflammation (31). Based on this evidence, dysregulated HOXA2 expression might exert anti-inflammatory effects during AS progression, particularly in response to vascular endothelial injury. However, the specific molecular mechanisms underlying this potential role require further exploration and validation.

This study utilized publicly available gene expression data from the GEO database and eQTL data to establish associations between genetic variants and gene expression levels. By integrating these resources, associations between exposures (gene expression) and outcomes (phenotypes) can be rapidly identified. Using eQTLs as IVs directly connects genetic regulation to gene expression, thereby strengthening the biological plausibility of causal inference (32). MR analysis mitigates confounding factors inherent in traditional observational studies by leveraging the random allocation of genetic variants during conception (10). Moreover, since genotypes are fixed at birth, this approach reduces reverse causation bias, where disease status could influence gene expression. By combining tissue-specific eQTL data with disease-specific expression profiles from the GEO database, this study facilitates the exploration of tissue-specific causal effects and disease heterogeneity. However, GEO data derived from diverse experimental platforms, sample processing protocols, and study designs may introduce batch effects or technical biases. To address this, stringent quality control measures, including batch effect correction, were implemented. Additionally, the eQTL data used in this study were predominantly based on European populations, limiting ancestral diversity. Future research should incorporate multi-ethnic cohorts to improve generalizability. To ensure analytical robustness, pleiotropic IVs were excluded, weak IVs were corrected, and variants in LD were removed. Two independent MR analyses were conducted in our study. The first analysis linked eQTLs to disease phenotypes to identify causal gene expression signatures. These candidate genes were then intersected with disease-associated DEGs from the GEO dataset to identify key genes of interest. A subsequent MR analysis was performed on these prioritized genes, requiring consistent directional effects across five complementary MR methods (e.g., IVW, MR-Egger, and weighted median). This dual MR framework minimizes bias from single-analysis limitations, significantly enhancing the reliability of causal inferences between genes and diseases, thereby strengthening the credibility of the theoretical model.

## 5 Conclusions

Through integrated multi-omics data and MR analysis, this study systematically identified CLEC5A and ISG20 as key risk genes for AS, with their elevated expression significantly associated with disease progression. Experimental validation demonstrated that ISG20 is specifically upregulated in ox-LDL-stimulated macrophages, likely promoting plaque development by modulating inflammatory responses and lipid metabolism. This study is the first to elucidate the dual role of ISG20 in AS pathogenesis, driving both lipid metabolic dysregulation and the amplification of inflammation, thereby providing experimental evidence for its potential as a therapeutic target. In contrast, CLEC5A primarily exacerbates inflammation through the activation of immune signaling pathways. These findings offer novel insights into the molecular mechanisms of AS and highlight the therapeutic potential of targeting these genes. Future research should focus on elucidating the functional mechanisms of these high-risk genes in AS progression and developing targeted inhibitors to assess their clinical viability. Additionally, expanding eQTL analyses to include diverse ancestral populations will enhance the generalizability of the identified genetic associations.

## Data Availability

The original contributions presented in the study are included in the article/**Supplementary Material**. Further inquiries can be directed to the corresponding author.
