# Supplementary material for: Causal inference of CLEC5A and ISG20 in atherosclerosis: integrating Mendelian randomization and eQTL evidence
Source: Front Immunol. 2025 Dec 3;16:1644135. doi: 10.3389/fimmu.2025.1644135 (PMC12708247; doi:10.3389/fimmu.2025.1644135)
Supplement: Supplementary file 1 [file DataSheet1.docx]

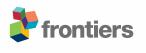


***Supplementary Material***

**Supplementary Figures**

**
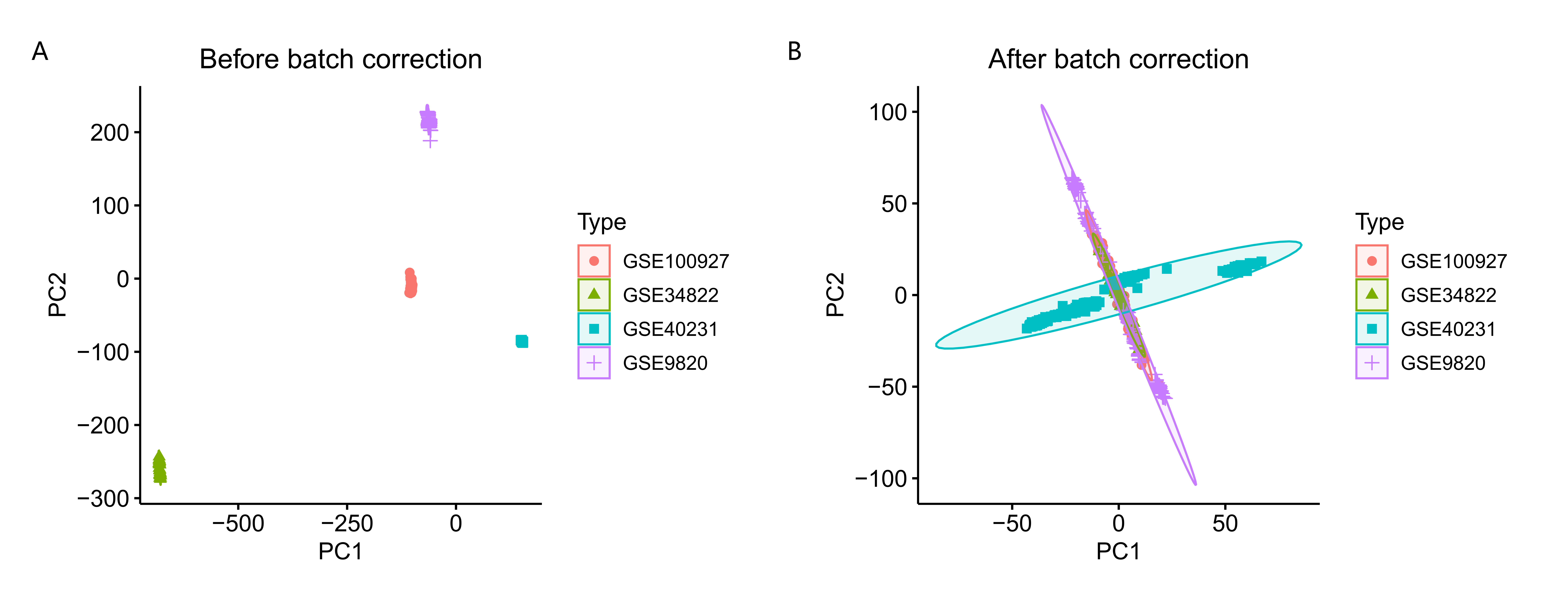
**

**Figure S1. Principal Component Analysis (PCA) of gene expression data before and after batch effect correction.** (A) PCA plot showing sample distribution prior to batch correction. Samples are colored by dataset of origin (GSE100927, GSE34822, GSE40231, GSE9820). Clear separation along PC1 indicates strong batch effects. (B) PCA plot after applying batch correction. The removal of technical variation results in improved overlap of samples across different datasets, indicating successful integration of the data.

Abbreviations:PCA, Principal Component Analysis; PC1, Principal Component 1; GSE, Gene Expression Omnibus Series (accession number for public genomic datasets).


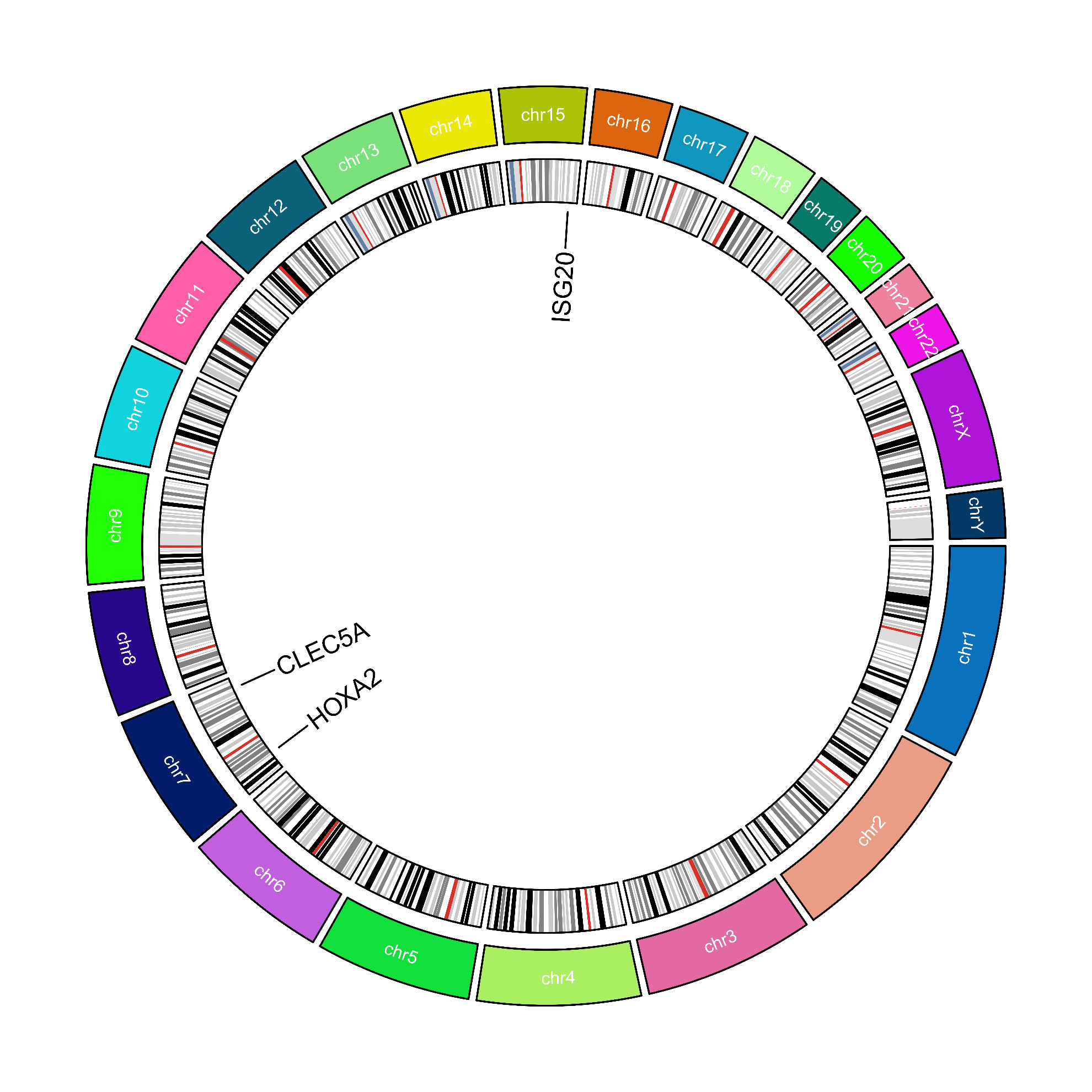


**Figure S2. Circos plot displaying the distribution and density of genomic features across all chromosomes.** The outer ring represents the ideograms of individual chromosomes (labeled chr13 to chr692). Inner tracks depict various genomic annotations, such as gene density (green), repeat elements (orange), and GC content (blue). Regions of high density are indicated by peaks in the corresponding track.

**Abbreviations:** **chr**, Chromosome;

**

**

**Supplementary Figure 3. Functional enrichment analysis of differentially expressed genes.** **(A) Bar plot of GO enrichment analysis.**Significantly enriched GO terms in Biological Process (BP), Cellular Component (CC), and Molecular Function (MF) are shown for three input genes (CLEC5A, ISG20, HOXA2). The bar plot is faceted by GO ontology, with bar height indicating the count of enriched genes and color representing the significance level (p-value or adjusted p-value). **(B) Bubble plot of GO enrichment analysis.** The same set of significantly enriched GO terms is visualized, with bubble size proportional to the number of enriched genes and color indicating the significance level (p-value or adjusted p-value). The x-axis represents the GeneRatio (ratio of enriched genes to total genes). **(C) Circular visualization of GO enrichment analysis.** The circplot displays GO terms in a circular layout. Outer ring: GO IDs; middle ring: number of background genes (pink) and number of selected genes (purple); inner ring: Rich Factor (green to red gradient). Colors distinguish BP (green), CC (yellow), and MF (purple). Legends indicate gene counts, Rich Factor, and −log10(Pvalue) ranges.

**Abbreviations:** GO, Gene Ontology; BP, Biological Process; CC, Cellular Component; MF, Molecular Function; GeneRatio, ratio of enriched genes to total genes; Rich Factor, ratio of enriched genes to background genes; Pvalue, p-value of enrichment; adjPval, adjusted p-value.

Supplementary Material

**
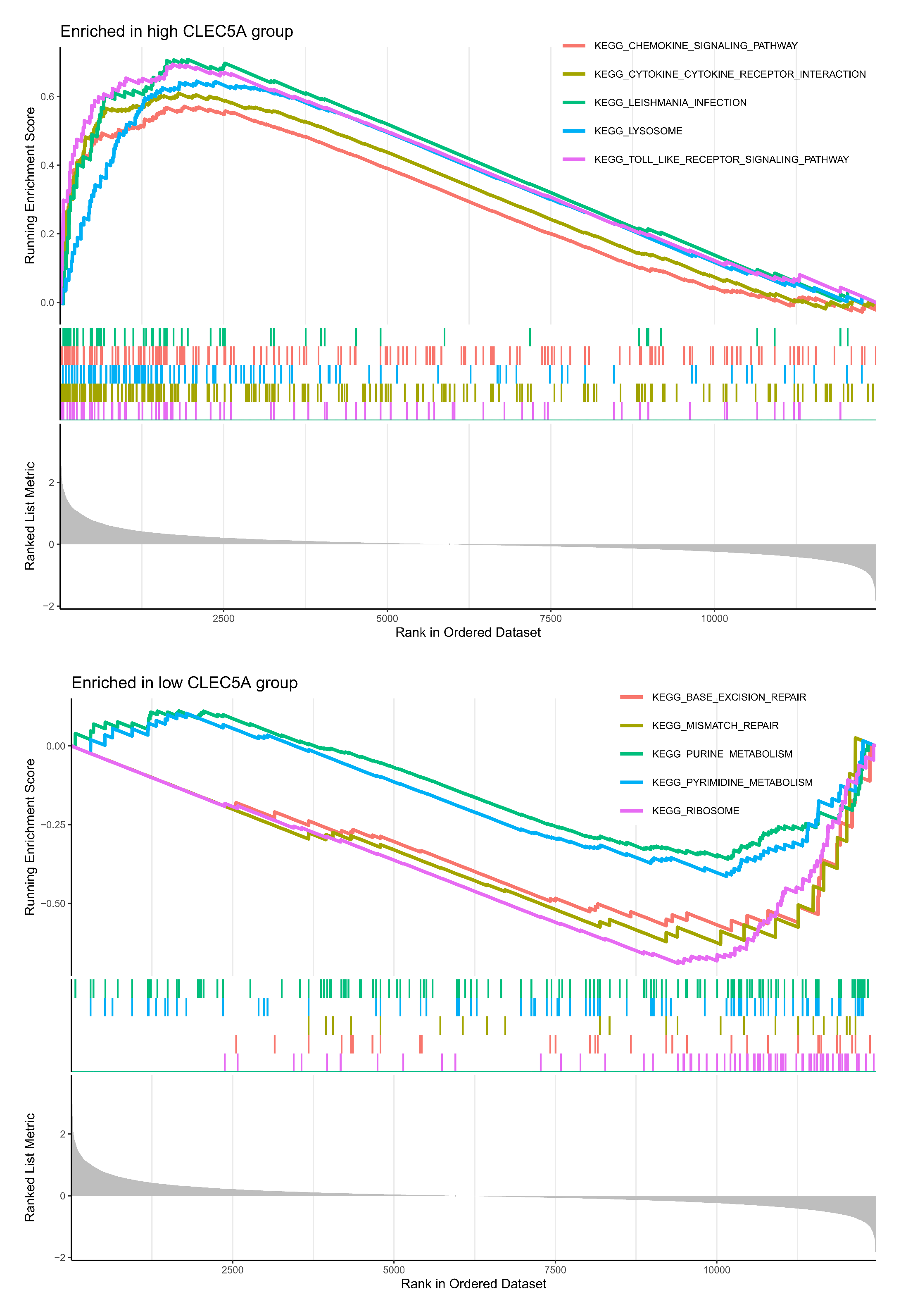
**

**Supplementary Figure 4.** **GSEA Pathway Enrichment Plots for High and Low CLEC5A Expression Groups.** Gene Set Enrichment Analysis (GSEA) shows pathways significantly enriched in samples with high expression of CLEC5A. The top plot displays the running enrichment score, while the bottom plot shows the ranked list metric. Enriched pathways include chemokine signaling, cytokine-cytokine receptor interaction, Leishmania infection, lysosome, and Toll-like receptor signaling pathways. In samples with low expression, key enriched pathways include base excision repair, mismatch repair, purine metabolism, pyrimidine metabolism, and ribosome. The running enrichment score (top) and ranked list metric (bottom) demonstrate the distribution of gene set enrichment across the ordered dataset.

**Abbreviations:** GSEA, Gene Set Enrichment Analysis; KEGG, Kyoto Encyclopedia of Genes and Genomes; ES, Enrichment Score; FDR, False Discovery Rate.

**
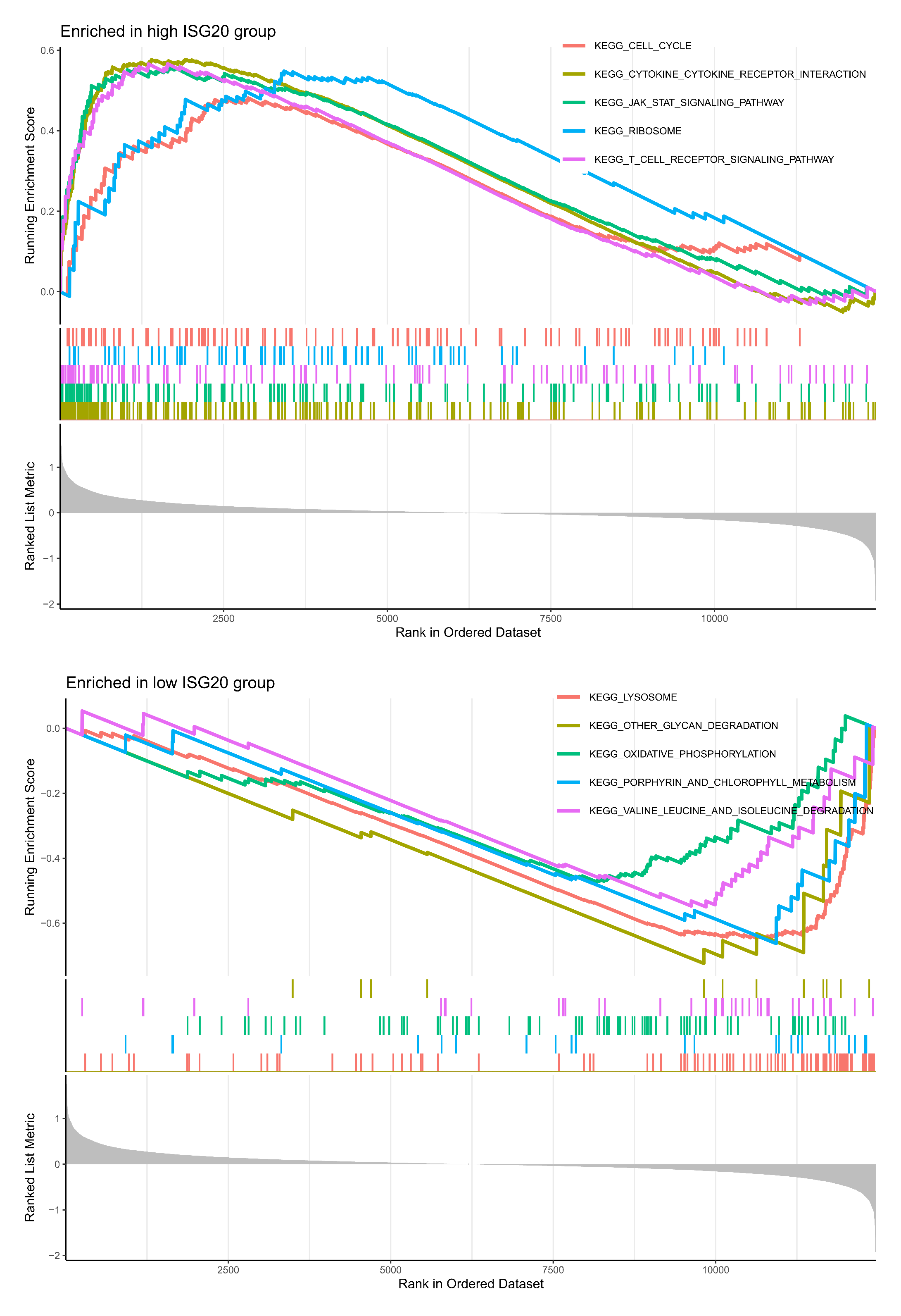
**

**Supplementary Figure 5.** **GSEA Pathway Enrichment Plots for High and Low ISG20 Expression Groups.** Gene Set Enrichment Analysis (GSEA) identifies pathways significantly enriched in samples with high ISG20 expression. Key enriched pathways include cell cycle, cytokine-cytokine receptor interaction, JAK-STAT signaling pathway, ribosome, and T cell receptor signaling pathway. In samples with low ISG20 expression, notable enriched pathways include lysosome, other glycan degradation, oxidative phosphorylation, porphyrin and chlorophyll metabolism, and valine, leucine and isoleucine degradation. The enrichment profile (top) and rank metric (bottom) demonstrate the position and magnitude of enrichment peaks within the ranked gene list.

**Abbreviations:** GSEA, Gene Set Enrichment Analysis; KEGG, Kyoto Encyclopedia of Genes and Genomes; ES, Enrichment Score; NES, Normalized Enrichment Score; FDR, False Discovery Rate

**
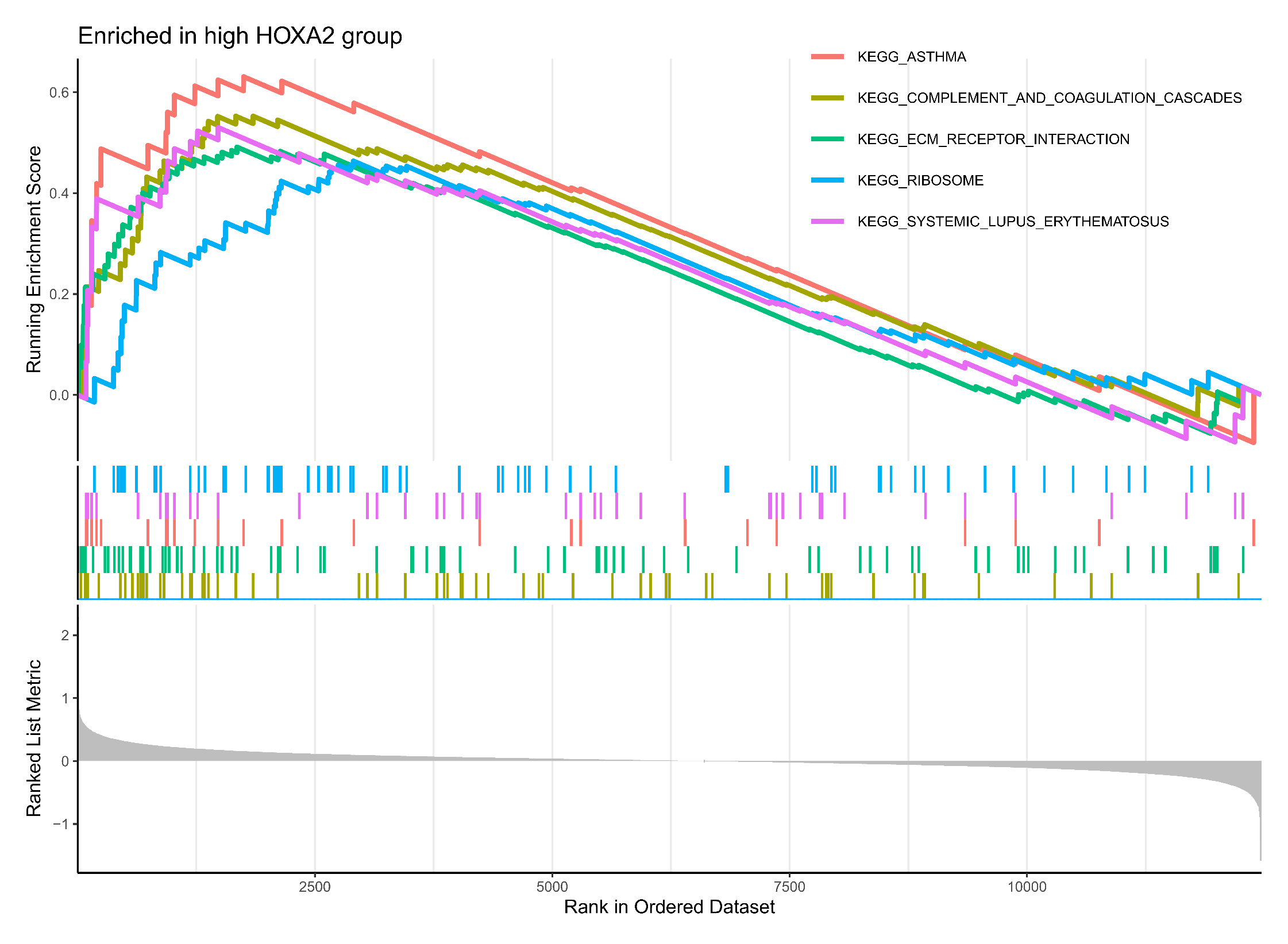
**

group. **Supplementary Figure 6. GSEA enrichment plot for the high HOXA2 expression group.** Gene Set Enrichment Analysis reveals pathways significantly enriched in samples with high HOXA2 expression. Key enriched pathways include asthma, complement and coagulation cascades, ECM-receptor interaction, ribosome, and systemic lupus erythematosus. The running enrichment score (top) demonstrates the cumulative enrichment pattern, while the ranked list metric (bottom) shows the position of core enriched genes within the ordered dataset.

**Abbreviations:** GSEA, Gene Set Enrichment Analysis; KEGG, Kyoto Encyclopedia of Genes and Genomes; ES, Enrichment Score; ECM, Extracellular Matrix.

**
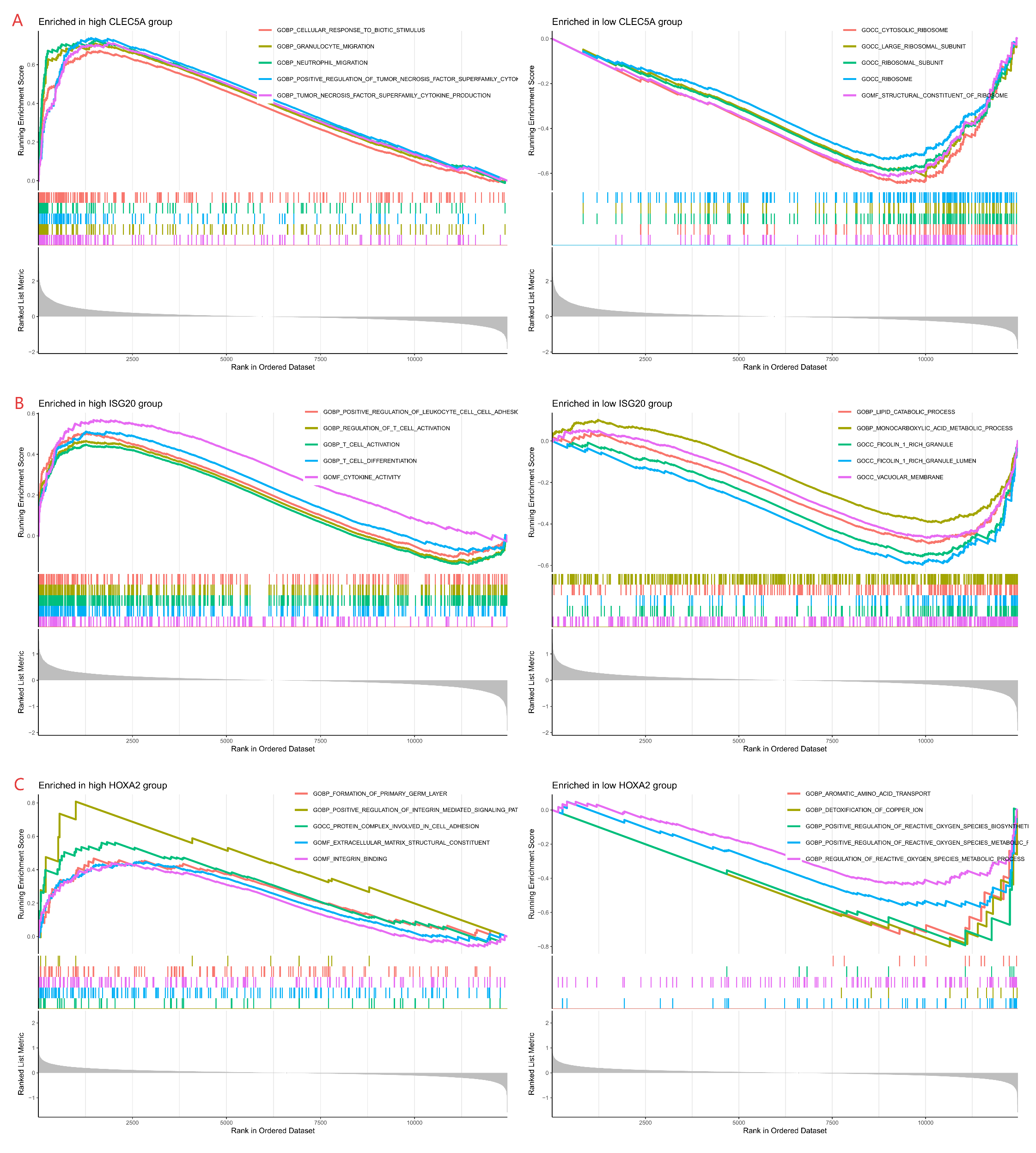
**

. **Supplementary Figure 7. Functional enrichment analysis of three key genes. (A)** Significantly enriched biological processes in the high CLEC5A expression group, including cellular response to biotic stimulus, granulocyte/neutrophil migration, and positive regulation of TNF superfamily cytokine production. Significantly enriched cellular components and molecular functions in the low CLEC5A expression group, primarily related to ribosomal structure (cytosolic ribosome, large ribosomal subunit) and function (structural constituent of ribosome). **(B)** Significantly enriched terms in the high ISG20 expression group include biological processes related to T cell activation and differentiation, positive regulation of leukocyte cell-cell adhesion, and molecular functions involving cytokine activity. In the low ISG20 expression group, enriched terms comprise lipid catabolic process, monocarboxylic acid metabolic process, and cellular components including ficolin-1-rich granule and vacuolar membrane. **(C) T**he high HOXA2 expression group shows enrichment in biological processes including primary germ layer formation and positive regulation of integrin-mediated signaling pathway, along with cellular components and molecular functions related to cell adhesion (protein complex involved in cell adhesion), extracellular matrix structural constituent, and integrin binding. The low HOXA2 expression group demonstrates enrichment in biological processes involving aromatic amino acid transport, copper ion detoxification, and regulation of reactive oxygen species metabolic processes. The running enrichment score (top) and ranked list metric (bottom) for each plot illustrate the distribution of gene set enrichment signals across the rank-ordered dataset.

**Abbreviations:** GSEA, Gene Set Enrichment Analysis; GO, Gene Ontology; GOBP, Gene Ontology Biological Process; GOCC, Gene Ontology Cellular Component; GOME, Gene Ontology Molecular Function; TNF, Tumor Necrosis Factor; ECM, Extracellular Matrix; ROS, Reactive Oxygen Species.
